# What can AI-TENG do for Low Abundance Biosensing?

**DOI:** 10.3389/fbioe.2022.899858

**Published:** 2022-05-05

**Authors:** Min Jiang, Shaoqiu Zheng, Zhiyuan Zhu

**Affiliations:** ^1^ Chongqing Key Laboratory of Nonlinear Circuits and Intelligent Information Processing, College of Electronic and Information Engineering, Southwest University, Chongqing, China; ^2^ The 28th Research Institute of China Electronics Technology Group Corporation, Nanjing, China

**Keywords:** artificial intelligence, triboelectric nanogenerators, biomolecules sensing, organic compounds, complex mixture cells

## Abstract

Biosensing technology helps prevent, diagnose, and treat diseases and has attracted more and more researchers in recent years. Artificial intelligence-based triboelectric nanogenerators (AI-TENG) are promising for applications in biosensors due to their myriad of merits, including high efficiency and precision, low cost, light weight, and self-powered. This article aims to show how artificial intelligence and triboelectric nanogenerators have been combined to develop biosensors. We first focus on the working principle of triboelectric nanogenerators and the method of combining them with artificial intelligence. Secondly, we highlight the representative research work of AI-TENG in biomolecules sensing, organic compounds, and complex mixture of cells. Finally, this paper concludes with a summary and prospect on the existing challenges and possible solutions in the application of AI-TENG to the field of biosensors.

## Introduction

The health status of a person is reflected by different physiological signals and monitoring health conditions in real-time can help prevent, diagnose and treat diseases. As a result, biosensors have been given more and more attention in recent years. Current biosensing systems rely on rechargeable batteries, which has largely limited the development of miniaturized and portable medical devices. Considering the increasing number of biosensors, providing sustainable energy sources will be an issue to be addressed (Wang X et al., 2021). Wang developed the first nanogenerator based on concentrating charge on the side and toward the bend, which initiates the current generation once released in a back-and-forth motion (Wang et al., 2007). Depending on the principle of operation, nanogenerators can be classified into two types: piezoelectric nanogenerators (PENG) and triboelectric nanogenerators (TENG), enabling self-powered biosensors to harness different forms of energy, including solar, thermal, mechanical, and biological energy. With their high sensing resolution, fast response, and flexibility, nanogenerators can be used in several applications, including biomedical devices (Feng et al., 2018; Sun et al., 2019; Khandelwal, 2020; Zhang et al., 2021), ocean wave energy harvesting (Jiang et al., 2015; Saadatnia et al., 2017; Wu et al., 2019), wind farms (Wang Y et al., 2021), and vehicle systems (Askari et al., 2019).

Apart from the advantages of nanogenerators for energy harvesting, they have shown great potential in facilitating interactions between humans and machines (Li et al., 2016; Ding et al., 2019; Xie et al., 2021) as combined with AI technologies. The application of AI in TENG can be divided into data collection and representation, algorithm determination, and model development (Salehi and Burgueno, 2018; Shi et al., 2020). In terms of data collection and representation, AI techniques need to be trained from existing data, so it is vital to maintain the accuracy and validity of the dataset (Mounet et al., 2018). Specific algorithms need to be identified to train the dataset after data collection and representation (Kalidindi et al., 2016). AI prediction models in TENG can either be rigid and simple [e.g., classic statistical linear regression models (Khorsand et al., 2020)] or complex and flexible [e.g., deep neural network models (Jiang et al., 2022)]. In TENG, classification algorithms can be used for analytical problems in design and manufacturing, and regression algorithms can solve probabilistic problems in application challenges (Jordan and Mitchell, 2015; Jiao, 2021).

This paper will report on the biosensor’s application of triboelectric nanogenerators combined with artificial intelligence (AI-TENG). Firstly, research work related to AI-TENG in low abundance biosensing is summarized, focusing on applying AI-TENG in biomolecules sensing (nano enzymes and nucleic acids), volatile organic compounds, and the regulation of complex mixed cells. Secondly, the existing challenges and possible solutions in the application of AI-TENG are discussed.

## AI-TENG Sensors for Low Abundance Biosensing

TENG-based AI devices are useful in advancing biosensing systems, enabling continuous and accurate acquisition of biosignals (Jiang et al., 2021). Analyzing this collected data through AI technology is expected to enhance diagnostic capabilities significantly, thus aiding the development of the next generation of digital biomedicine (Stuart et al., 2021).

### Detecting Biomolecules

**FIGURE 1:**
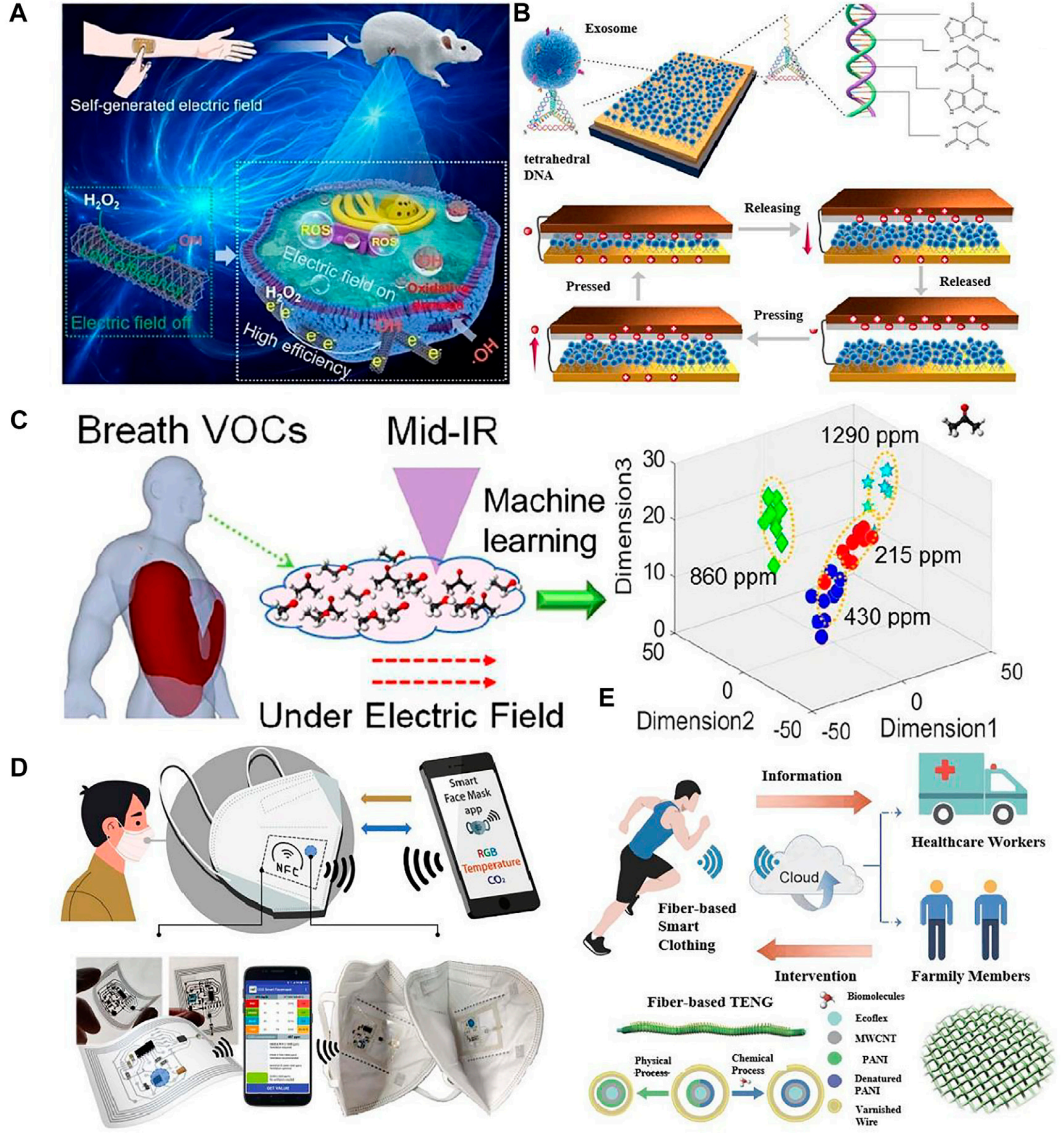
Biosensing applications of AI-TENG in previous reports. **(A)** The schematic diagram of the human self-powered catalysis-promoting system (Yao et al., 2022). **(B)** The schematic diagram of the triboelectric sensor for direct quantification of exosomes (Miao et al., 2022). **(C)** The schematic diagram of the Machine-learning-assisted and plasma enhancement mid-IR methodology for VOC healthcare diagnosis (Zhu et al., 2020). **(D)** The schematic diagram of the smart facemask for wireless CO_2_ real-time determination (Escobedo et al., 2022). **(E)** The schematic diagram of the developed smart clothing applied in a closed-loop system (Zhao et al., 2022).

Combining AI-TENG with biotechnology has received increasing attention in recent years, and this technology has a wide range of applications in the medical field. Yao et al. developed a human self-powered catalysis-promoting system, TENG-CatSystem, to improve catalytic cancer therapy Yao et al. (2022). TENG-CatSystem is composed of a self-powered TENG and a one-dimensional ferriporphyrin covalent organic framework coated on a carbon nanotube (COF-CNT). The peroxidase-like activity of COF-CNT was increased approximately four-fold under the electric field provided by the wearable TENG (Figure 1A). In addition, the treatment process and results can be displayed at the computer terminal using AI technology, which greatly enhances the treatment cycle and treatment results. In order to achieve a simple and accurate determination of exosomes, Miao et al. have developed a novel triboelectric sensor based on tetrahedral DNA modifications Miao et al. (2022). Upon interaction with exosomes, the contact area of the top and bottom portions of the TENG device increases significantly, enabling direct quantification of exosomes and ultrahigh sensitivity of the sensor. By combining with AI techniques, exosomes can be monitored in real-time and intuitively after changing the DNA sequence, and this AI-TENG sensing strategy can analyze most types of targets (Figure 1B).

The AI-TENG biomolecular detection method enables self-powered and real-time monitoring in biotechnology, greatly simplifying the external equipment of biosensors and improving detection efficiency. Therefore, AI-TENG has a wide range of promising applications in the field of biomolecules biosensor systems.

### Detecting Organic Compounds

As a biological monitoring tool for human health, volatile organic compounds (VOCs) can be used as important biomarkers for healthcare monitoring and early diagnosis of diseases. Zhu et al. reported a plasma-enhanced infrared absorption spectrum with fast response, accurate quantification, and good selectivity using a plasma-induced ultra-high electric field to enhance the vibration of molecules and improve light-matter interactions Zhu et al. (2020). The types of VOCs and their concentrations can be well quantified from the wavelength and intensity of the plasma-enhanced spectral signals (Figure 1C). In addition, ML algorithms visualized the relationship between different VOCs in the mixture, demonstrating the feasibility of VOC identification for simulated patients. A machine learning enhanced ion mobility analyzer with a triboelectric-based ion generator is also reported by Zhu et al., which provides good ion mobility selectivity and VOC identification in small devices and non-strict operating environments Zhu et al. (2021). By extracting specific features automatically from ion mobility spectroscopy data with an ML algorithm that significantly improves the detection capability of the TENG VOC-based analyzer.

In order to mitigate the rapid global spread of severe acute respiratory syndrome coronavirus 2, the design and evaluation of a battery-free, wearable mask is an effective solution. Escobedo et al. report a sensing platform for real-time measurement of gaseous CO_2_ in the FFP2 mask Escobedo et al. (2022). Moreover, AI technology has developed a bespoke smartphone application for wireless power supply, data processing, alarm management, results display, and sharing (Figure 1D). Daily activity and monitoring performance tests demonstrate their utility in non-invasive, wearable health assessment and potential applicability in pre-clinical studies and diagnostics.

The artificial intelligence algorithm provides a large amount of information on parameters for detecting VOCs and the TENG provides a self-powered supply for the monitoring system. Thus, AI-TENG provides an effective solution for building a complete organic compound monitoring system.

### Detecting Complex Mixture Cells

The detection of complex mixtures in the body’s cells or tissues provides a timely indication of the body’s health status (Teng et al., 2022). Zhao prepared stretchable fiber TENG (F-TENG) by sequentially coating multi-walled carbon nanotubes and polyaniline onto Ecoflex fibers (Figure 1E). They detected glucose, lactate, and creatinine concentrations (Setiyorini et al., 2022) in sweat through enzyme modifications (glucose oxidase, lactate oxidase, creatinine oxidase). Moreover, by connecting the F-TENG to a wireless communication device, the detected information can be transmitted to the cloud in real-time, and AI algorithms can process the data to build a self-powered closed-loop health monitoring system (Zhao et al., 2022). Li has developed a bio-nanogenerator consisting of highly discrete piezoelectric (Luo et al., 2022) fibers to achieve precise electronic modulation of single regional cells. The sensor can mimic the complex structure highly. The self-generated electronic function of natural collagen extracellular matrix (ECM) nanofibers in terms of three-dimensional structure and electrical properties (Li et al., 2021) enables *in situ* simulated electrophysiological stimulation of cells and tissues and the construction of complex microenvironments, significantly promoting the activity and functional expression of a wide range of cells (neuronal cells, hepatocytes, bone marrow mesenchymal stem cells, etc.) and tissues (liver, peripheral nerves).

AI-TENG uses a large amount of statistical real-time feedback data to train a mathematical model with a specific structure containing unknown parameters to generate a detector for complex biological cells containing the statistical features inherent in the training data. This approach greatly reduces the cost of testing equipment and increases testing efficiency.

## Discussion

As illustrated by the three examples, AI-TENG is promising for applications in low abundance biosensing due to its myriad of merits, including high efficiency and precision, low cost, light weight, and self-powered. However, there are many challenges in these applications. Firstly, it is necessary to develop effective sensing systems that are cheap, reliable, and fully functional in the long term. As data from different sources may be fused for biosensors, fully fault-tolerant self-powered sensing systems are needed to reduce the influence of faulty measurements on the entire output of the sensing system. This can be achieved by using embedded software that can provide a remedy in the event of a faulty measurement. Secondly, most of the works related to the integration of AI-TENG technologies are still at the proof-of-concept stage and have yet to be enhanced for real-world applications. Scaling these applications to larger data sets will present unique challenges. Finally, the use of AI tools and the increased flow of data from continuously operating sensing systems requires specific hardware for data processing, model training, and evaluation. Therefore, combining 5G networks, cloud computing, AIoT devices, and the development of tiny machine learning could help address challenges.

There is exciting potential for low abundance biosensing to harness the powerful products provided by AI-TENG. It plays an important role in the detection of biomolecules, organic compounds, and complex mixture cells. On the one hand, due to the unique sensing and energy harvesting capabilities, TENG can facilitate the development of intelligence devices focused on intelligent self-powered sensing systems. On the other hand, the AI algorithm automatically extracts the intrinsic features of the target through the internal network structure. It builds a stable combination of features through a low-level to high-level abstraction process, weakening the subjectivity of manual feature selection and saving a lot of time and workload. Combining triboelectric nanogenerators with artificial intelligence in biosensing will result in the detection systems having the ability to learn, think, and make decisions in real-time without the need for an external power source. Therefore, AI-TENG has transformed many sensing systems in different applications, such as providing the intelligent electronics needed for biosensing devices. Under such conditions, the title question to ask “what can AI-TENG do for low abundance biosensing?”, these answers have exciting prospects for the coming years.
